# American and British Doctors and the G.M.C.

**Published:** 1917-12-08

**Authors:** 


### The Hospital

The Workers' Newspaper of Administrative Medicine and Institutional Life, Administration, National Insurance and Health.

No. 1644, Vol. LXIII. SATURDAY, DECEMBER 8, 1917. PRICE ONE PENNY

### AMERICAN AND BRITISH DOCTORS AND THE G.M.C.

As we have remarked on previous occasions, the work of the General Medical Council, at least for the most part, is not of a nature to make a pressing appeal to popular attention, but the Council has important responsibilities, and these cover a wide range of interest. The burden which falls upon the President of the Council is a very heavy one, and there is good evidence that the present occupant of the Chair, Sir Donald MacAlister, gives himself in a thorough and statesmanlike fashion to the discharge of his varied obligations. Medical legislation throughout the whole of the Empire may create delicate questions for adjustment and reciprocity. The force of this last-mentioned consideration has illustration at the present moment. For reasons which are obvious, Western Europe, including our own country, is looking with interest and expectation to the arrival from the United States of fully equipped armies ready to take a share in the struggle for victory over the common enemy.

As is well known, a medical contingent, a sort of advance-guard of the combatant forces, arrived in this country early in the summer. The arrangement was intended to serve a double purpose. First, by taking over work in military hospitals the American doctors were able to set free a certain number of our own men for civil practice, while, in the second place, the doctors gained the opportunity of experience in the special forms of medical and surgical work which the war has created. With the arrangement and its purposes there was general sympathy, and to their American colleagues the profession have extended a sincere and unrestrained welcome.

Yet, strange as it may seem, it is none the less true that in a strictly legal sense these gentlemen were destitute of the technical status necessary to hold medical appointments in this country. For such appointments the name of the holder must be on the Medical Register. But the names of American graduates cannot be entered on this Register because between this country and the United States there is no registration reciprocity. The Medical Council has a certain power to register persons holding a foreign qualification, but this only extends to countries which exercise a similar custom in regard to British registered practitioners. Now there is no Federal legislation in the United States authorising reciprocity with this country, and hence neither the General Medical Council, nor even the King in Council, has the power to admit American medical practitioners to the British Register. The status of those who are working here is strictly that of unregistered practitioners, and it cannot be altered except by legislation, and legislation, on such a subject, clearly cannot be arranged before the termination of the war. In time it will come, without doubt, for the principle of accepting for the British Register diplomas obtained outside the United Kingdom has been widely recognised, as is shown by the inclusion in the official list of something like a thousand practitioners who have acquired their qualifications not in this country but at Colonial or foreign medical schools. But not unnaturally the law requires a *quid pro quo* in the shape of reciprocity, and as this does not exist between Britain and America our Transatlantic colleagues cannot be included. When peace comes again this position must be reviewed, and we hope a prompt proposal will come from this side. There are, we know, certain difficulties, but it is high time these were overcome. Formalities and technicalities are apt to have an irritating quality, and there are abundant reasons why, so far as possible, we should get rid of them.

If in the meantime the American doctors are giving us some help and service in our military hospitals, and are thus indirectly relieving the acute medical claims of the civil population, it is necessary to note that this help cannot be long continued. Ere long they will be summoned to take their places with the American forces in France, and our military hospitals here must revert to the care of British practitioners. Some of these feel—and justly so—that they have not been very well treated, but none of them, we are sure, will allow a sense of resentment to interfere with the claims of our sick and wounded soldiers. The officials may have been tactless and "superior," and in these columns we have not hesitated to say some hard things of them. But it is the fighting men of whom we have to think, and whatever else fails help to these must certainly not be stinted. Therefore, when our American colleagues cross the Channel, carrying with them all our good wishes, our own men must be ready to take their places, and we are sure that they are fully ready to do so. This will mean a further invasion of the measure of medical help available for the civil population, and the issue is a serious one. Here we have urged, on more than one occasion, that the existing degree of exemption from military service extended to medical students must be enlarged, and we are glad to know that the Minister of National Service has decided to make a move in this direction. His intention is to adopt regulations which will secure the annual admission to the Register of about one thousand new practitioners. This is, indeed, a minimum figure in view of the wastage revealed by the number of names removed from the Register in the three years 1911-1913, with an annual average of 620, while in 1916 the number was 983. The figures indicate a serious position, and the General Medical Council has discharged a public duty in urging that recruiting for the Army among medical students be so modified as to secure for the country the medical service which is necessary.

